# Yttrium-90 Radioembolization of Hepatic Metastases from Colorectal Cancer

**DOI:** 10.3389/fonc.2014.00120

**Published:** 2014-07-25

**Authors:** Mihir Raval, Dinesh Bande, Anil K. Pillai, Lawrence S. Blaszkowsky, Suvranu Ganguli, Muhammad S. Beg, Sanjeeva P. Kalva

**Affiliations:** ^1^Department of Hospital Medicine, Essentia Health, Fargo, ND, USA; ^2^Department of Hospital Medicine, Sanford Health, Fargo, ND, USA; ^3^Roger Maris Cancer Center, Fargo, ND, USA; ^4^Department of Internal Medicine, University of North Dakota, Fargo, ND, USA; ^5^Harold Simmons Cancer Center, University of Texas Southwestern Medical Center, Dallas, TX, USA; ^6^Interventional Radiology, University of Texas Southwestern Medical Center, Dallas, TX, USA; ^7^Massachusetts General Hospital Cancer Center, Boston, MA, USA; ^8^Department of Hematology and Oncology, Massachusetts General Hospital, Boston, MA, USA; ^9^Department of Medicine, Harvard Medical School, Boston, MA, USA; ^10^Section of Interventional Radiology, Department of Imaging, Massachusetts General Hospital, Boston, MA, USA; ^11^Department of Radiology, Harvard Medical School, Boston, MA, USA; ^12^Division of Hematology and Oncology, Department of Medicine, University of Texas Southwestern Medical Center, Dallas, TX, USA

**Keywords:** colorectal cancer, liver metastases, radioembolization, Yttrium-90 microspheres, Y-90 therapy, selective internal radiation therapy

## Abstract

Liver metastases from colorectal cancer (CRC) result in substantial morbidity and mortality. The primary treatment is systemic chemotherapy, and in selected patients, surgical resection; however, for patients who are not surgical candidates and/or fail systemic chemotherapy, liver-directed therapies are increasingly being utilized. Yttrium-90 (Y-90) microsphere therapy, also known as selective internal radiation therapy (SIRT) or radioembolization, has proven to be effective in terms of extending time to progression of disease and also providing survival benefit. This review focuses on the use of Y-90 microsphere therapy in the treatment of liver metastases from CRC, including a comprehensive review of published clinical trials and prospective studies conducted thus far. We review the methodology, outcomes, and side effects of Y-90 microsphere therapy for metastatic CRC.

## Introduction

The liver is the most common site of metastases from colorectal cancer (CRC). Approximately 20% of patients are found to have distant metastases at the time of CRC diagnosis with approximately 40% to the liver (1, 2). An additional 35–60% of patients develop liver metastases in the course of the disease. Presence of liver metastases portends a poor prognosis. Of the patients who develop liver metastases, one-fifth dies with liver metastases only. In the absence of treatment, the median survival is approximately 4–6 months. Surgical resection of liver metastases provides the best 5-year survival, in the order of 40%, and is possible if the disease is localized or downgraded with systemic chemotherapy and a safe surgical margin can be achieved without significant post-operative liver dysfunction (3–5). Only 10–20% of patients who present with liver metastases from CRC are surgical candidates (2–5). The role of standard external beam radiation has been limited due to poor tolerance of the normal liver tissue to radiation. As such, palliative chemotherapy had for many years been the only option for the majority of patients with liver metastases from CRC. The fluropyrimidine 5-fluorouracil has been used in the management for several decades. The introduction of several new cytotoxic agents, including oxaliplatin, irinotecan, and targeted therapies including VEGF and EGFR inhibitors, has resulted in substantial improvement in median survival, which now exceeds 2 years (6–9).

Since the majority of CRC metastases occur in the liver and the benefit from systemic chemotherapy has been modest until recently, selective hepatic arterial chemotherapy through arterial infusion of 5-fluorouracil has been practiced with mixed results (10, 11). Such therapy is based on the principle that the metastases receive the majority of blood flow from the hepatic artery (12) and direct arterial infusion of chemotherapeutic drugs would result in higher tumoral concentration of the drugs compared to those achieved with intravenous administration due to high first pass hepatic extraction of the drugs (13). New regimens of systemic chemotherapy have virtually replaced hepatic artery infusion chemotherapy due to better results (6). However, there has been resurgence of hepatic artery directed therapies through the use of microparticles that either carries radiation (radioembolization) or chemotherapeutic drugs (chemoembolization) (14). In this article, we present the rationale, treatment aspects, and results of radioembolization in the treatment of hepatic colorectal metastases.

## Rationale of Radioembolization Therapy

Radioembolization with Yttrium-90 (Y-90) microspheres is based on the same principle as that of hepatic artery infusion chemotherapy. The hepatic artery provides the primary vascular supply to the tumor with >80% of the blood flow to the tumor being derived from the hepatic artery whereas the normal liver parenchyma receives the majority of its blood flow via the portal vein (12). In addition, the microvascular density of the hepatic tumors is 3–200 times higher than the surrounding normal liver parenchyma (15) leading to a higher localized entrapment of microparticles in the tumor when the microparticles are infused through the hepatic artery. CRC cells are highly radiosensitive and do not demonstrate any cross-resistance to radiation despite being chemotherapy-refractory. Additionally, radiation works synergistically when used with radiation-sensitizing chemotherapeutic drugs. Unlike external beam radiation, which is limited to treatment of small number of focal tumors due to concerns of liver toxicity with multi-focal disease, selective infusion of Y-90 microspheres allows selective “inside-out” radiation of multi-focal tumors through selective localization of the particles in the tumor vessels and localized emission of beta radiation in the tumor environment (14). Because of this, Y-90 radioembolization is also known as “selective internal radiation therapy” (SIRT) or intra-arterial brachytherapy.

## Yttrium-90 Microspheres

The first intra-arterial infusion of Y-90 radio-isotope for treatment of pancreatic and primary liver cancer was reported in 1965 (16). Since that time, there have been significant advances in the production, distribution, and administration of these microspheres. Currently, there are two commercially available Y-90 labeled microsphere preparations – TheraSpheres (BTG International Canada Inc, Ottawa, ON, Canada) and SIR-spheres (Sirtex Medical Inc., Sydney, NSW, Australia). Both microspheres measure 20–60 μm in size (TheraSpheres 20–30 μm and SIR-spheres, 20–60 μm), and are designed to carry Y-90. Y-90 is a beta radiation emitter, with a half-life of 64.1 h and an average energy of 0.94 MeV. The radiation has a range of 1.1 cm (average 2.5 mm in the tissues) from the source, and 94% of the dose is delivered within the first 11 days following administration (14). The microspheres remain embedded in the vascular bed permanently without undergoing any physical or chemical change. The major differences between the two preparations are the density and specific activity (radiation dose per microsphere) of the particles, and their FDA approval (17, 18). TheraSpheres are made of glass and Y-90 is embedded within the glass matrix leading to higher density and specific activity (2500 Bq) of the preparation. SIR-spheres are made of resin and Y-90 is coated on the surface of the particle leading to low density and low specific activity (50 Bq). The FDA approved TheraSpheres under the category of humanitarian device exemption for use in patients with unresectable hepatocellular carcinoma requiring on site institutional review board (IRB) oversight. SIR-spheres received FDA approval in 2002 as a brachytherapy device for treatment of unresectable hepatic metastases from CRC with adjuvant hepatic artery infusion of floxuridine.

## Administration of Yttrium-90 Microspheres

A successful intra-tumoral administration of Y-90 microspheres involves careful patient selection, assessment of the hepatic arteries and hepato-pulmonary shunt, dose calculation, and intra-arterial delivery of the microspheres.

### Patient selection

Careful evaluation for optimal patient selection is required to optimize the outcomes and limit toxicities of Y-90 radioembolization. The recommended selection criteria are listed in Table 1 (19). Patients with poor performance status, limited hepatic reserve, and extensive multi-organ metastases are poor candidates for this therapy. At many centers, baseline evaluation is performed with PET-CT to detect extra-hepatic disease and to assess the volume and biological activity of the tumor.

**Table 1:** **Patient selection criteria for Yttrium-90 radioembolization of hepatic metastases from colorectal cancer**.

**TUMOR SPECIFIC**
Liver metastases not eligible for surgery or local ablative therapy
Failed first line systemic chemotherapy (unless planning for concomitant systemic chemotherapy)
No significant hepato-pulmonary shunt (which results from presence of large intra-tumoral arteriovenous shunts). Radioembolization is not performed if the radiation dose to the lung exceeds 30 Gy per treatment
Absent or minimal extra-hepatic metastases
**LIVER SPECIFIC**
Relatively preserved liver and kidney function – serum bilirubin <2 mg/dL, serum creatinine <1.8 mg/dL; platelet count >50,000/μL
Preserved hepatopetal flow in the main portal vein
No risk of non-target delivery of the microspheres during hepatic arterial infusion
No prior radiation to the liver
**PATIENT SPECIFIC**
ECOG performance status of 2 or less
Life expectancy of >6 weeks

### Assessment of hepatic arteries and hepato-pulmonary shunt

Selective intra-arterial infusion of Y-90 microspheres requires careful angiographic evaluation of the hepatic arteries to plan for lobar or segmental delivery of the microspheres, to isolate hepatic perfusion to a few arteries (so as to minimize the number of infusions or to minimize risks of extra-hepatic delivery) and to pro-actively exclude the hepatic artery branches (such as the gastric or duodenal arteries) that are at risk of non-targeted delivery of the microspheres. The hepatic arterial branches that are at risk of non-target delivery are occluded (embolized) with coils.

The hepato-pulmonary shunt can lead to non-target deposition of Y-90 microspheres in the lung. Some of the microspheres escape the liver through large (>30 μm in diameter) intra-tumoral arteriovenous shunts and get entrapped in the pulmonary capillaries. The degree of shunting is estimated through intra-arterial infusion of Tc-99 m macro-aggregated albumin (4–5 mCi) and subsequent measurement of the lung and liver activity on a gamma camera. Presence of hepato-pulmonary shunt *per se* is not a contra-indication for treatment with Y-90 microspheres as long as the radiation dose per treatment to the lung does not exceed 30 Gy or the cumulative dose to the lungs is within 50 Gy.

### Dose calculation

Dose calculation is based on the tumor volume in the liver (20). Two methods popularly used include the empiric method and the body surface area (BSA) method. In the empiric method, 2 GBq of Y-90 dose is given for a tumor volume occupying <25% of the liver, 3 GBq if the tumor volume is 25–50% of the liver volume, and 5 GBq if the tumor volume exceeds >50% of the liver volume. BSA method takes in to consideration of the BSA, liver volume, and tumor volume and is tailored to the patient and the volume of the liver treated. The calculated dose in gigabecquerel is [(BSA − 0.2) + {Tumor volume/(Tumor volume + Liver volume)}]. Many centers in the US follow BSA method for dose calculation (19, 21, 22). Treatment based on the dose calculated by the empiric method (especially when the tumor volume exceeds >25%) results in very high, often fatal, radiation dose to the liver and is not recommended. The dose calculations for TheraSpheres are based on partition model with an intent to deliver 80–120 Gy of radiation dose to the treated volume of the liver.

### Infusion of Yttrium-90 microspheres

Selective infusion of Y-90 microspheres is achieved through closed circuit delivery using proprietary delivery device that is specific for each company. Both lobar and whole liver infusions are practiced; authors prefer to use lobar or segmental infusions over whole liver infusions in order to limit hepatic toxicities. Post infusion imaging of the liver through SPECT or PET is often performed (though not required) to assess the hepatic uptake pattern of the microspheres.

### Patient follow-up and subsequent treatments

**Figure 1:**
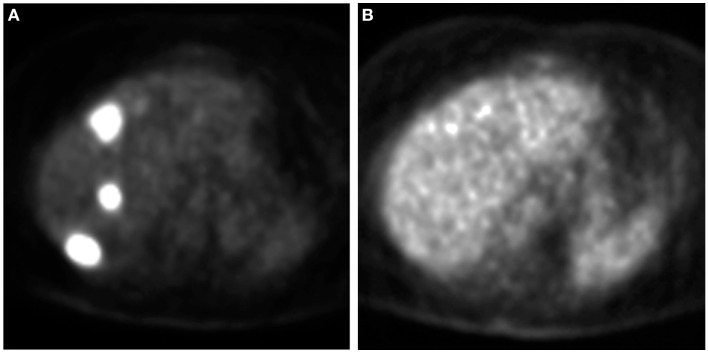
**Assessment of response to Y-90 therapy on PET**. A PET scan **(A)** obtained prior to Y-90 therapy demonstrates three FDG avid lesions. A repeat PET scan **(B)** obtained 6 weeks after Y-90 therapy shows no FDG avid lesions suggesting complete metabolic response.

**Figure 2:**
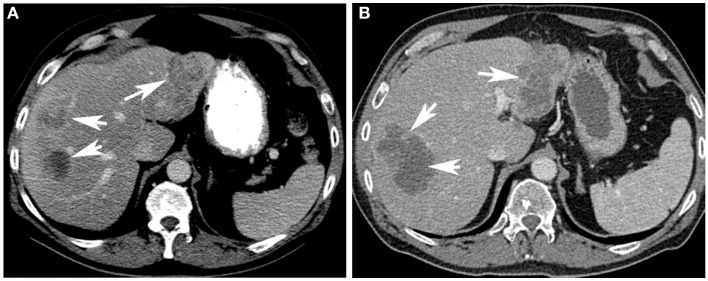
**Assessment of response to Y-90 therapy on CT**. A contrast enhanced CT scan **(A)** of the liver demonstrates hypo-attenuating lesions (arrows) in the liver. A repeat contrast enhanced CT scan **(B)** obtained 6 weeks after Y-90 therapy shows very low attenuation of the lesions (arrows). Even though the lesions size has not significantly changed, the development of very low attenuation on post-treatment scan suggests response to Y-90 therapy.

When not contraindicated, some centers administer systemic 5-fluorouracil before and after Y-90 therapy. Toxicities are assessed at 1 and 4 weeks following Y-90 therapy. In the presence of bilobar disease, Y-90 radioembolization of the other lobe is performed at 4–6 weeks from initial therapy. A follow-up PET-CT is performed at 6 weeks after second treatment. There are no standardized guidelines on timing of follow-up imaging studies to assess tumor response. PET has been shown to be useful in assessing response and guiding further treatment (Figure 1); the role of CT attenuation change of the tumor as a surrogate marker of response is currently being explored (Figure 2) (23–25). Subsequent imaging assessment is performed at 3 month intervals with PET-CT. Additional Y-90 infusions are performed (up to two treatments per lobe) if tumor recurs.

### Toxicities

Most patients report fever, lethargy, decreased appetite, and fatigue following therapy. Uncommon serious adverse events include radiation-induced gastric ulcers, lymphocytopenia, jaundice, cholecystitis, lung toxicity, hepatic abscess, radiation hepatitis, and liver failure (26–29). Patients are advised to take proton pump inhibitors to prevent gastrointestinal side effects. Use of peri-procedural steroids to prevent development of fatigue or chronic liver injury is also described (29). The hepatic injury from Y-90 appears to be secondary to development of portal triaditis (29).

## Outcomes

Given that Y-90 radioembolization for hepatic colorectal metastases has been used at various stages of the disease and with or without systemic or regional chemotherapy, the outcomes of this therapy are described in this manuscript according to the timing of this therapy and systemic chemotherapy.

### Yttrium-90 radioembolization as first line therapy

The first prospective study that led to FDA registration of Y-90 microspheres (SIR-spheres) for treatment of hepatic metastases from CRC included 74 patients who were randomized to receive hepatic arterial chemoinfusion (HAC) of floxuridine and single whole liver Y-90 therapy through hepatic artery access port or HAC of floxuridine alone (Table 2) (30). The final analysis included 70 patients. There was significantly higher objective response rate in patients who received Y-90 microsphere therapy in addition to HAC compared to those who received HAC alone (Response rate based on WHO criteria at 3 months was 44.4 vs. 17.6%, *p* = 0.01) (30). More patients in the Y-90 group demonstrated 50% or more reduction in elevated CEA levels following therapy (72.2 vs. 47.1%, *p* = 0.004) (30). The median time to tumor progression (TTP) was longer in the group that received Y-90 (15.9 vs. 9.7 months). Though a trend for a survival advantage in this group was observed, it did not reach statistical significance. Authors also reported 3.1 times (95% CI: 1.1–8.8) higher risk of death from progression of liver metastases in patients who received HAC alone. There were no significant differences in the incidence of Grade 3 or 4 toxic events in either group. This study is significant in that it demonstrated safety of Y-90 therapy when combined with HAC of floxuridine and such addition definitively improved objective response rate and TTP in predominately chemo-naive patients. However, the study failed to demonstrate any significant survival advantage of such combination therapy. This may be due to the small number of patients enrolled in this study. Secondly, the study randomized patients to HAC with floxuridine, which is rarely used as first line therapy. The first line chemotherapy currently employed includes a combination of 5-fluorouracil, leucovorin plus oxaliplatin, or irinotecan (FOLFOX or FOLFIRI) with or without biological agents such as bevacizumab.

**Table 2:** **Yttrium-90 radioembolization as first line therapy for hepatic colorectal metastases**.

Author	No. of patients	Trial design	Treatment	Response	Survival	Complications
Gray et al. (30)	74 (only 70 included in analysis)	Prospective, phase III, randomized	Y-90 radioembolization *plus* hepatic arterial chemoinfusion (HAC) with floxuridine (*n* = 36) vs. HAC with floxuridine (*n* = 34)	Response (CR + PR): 44 vs. 17.6% (*p* = 0.001) response (CEA): 72 vs. 47% (*p* = 0.004)	TTP: 15.9 vs. 9.7 m (*p* = 0.001); 1, 2, 3, and 5 year survivals: 72, 39, 17, 3.5 vs. 68, 29, 6.5, and 0%; median survival: 17 vs. 15.9 m (*p* = 0.18)	Higher incidence of grade 3 elevation of alkaline phosphatase in the group that received Y-90 plus HAC; no overall difference in the incidence of grade 3–4 toxicities among the two groups
Van Hazel et al. (31)	21	Prospective, phase II, randomized	Y-90 radioembolization *plus* systemic 5-fluorouracil and leucovorin (*n* = 11) vs. systemic 5-fluorouracil and leucovorin (*n* = 10)	Best confirmed response: 8PR, 3SD vs. 0PR, 6SD, 4PD (*p* < 0.001)	Median survival: 29.4 vs. 12.8 m (*p* = 0.02). PFS: 18.6 vs. 3.4 m	Grade 3 and 4 toxicity events: 13 vs. 5; No difference in quality of life
Sharma et al. (32)	20	Prospective, single arm; patients with unresectable disease and chemo-naive	Y-90 radioembolization *plus* FOLFOX	18PR, 2SD	TTP: 12.3 m in patients with EHD, 14.2 m in patients without EHD. Median PFS: 9.3 months	Grade 3–4 neutropenia in 12. Gastric ulcer in 1. Grade 3 hepatotoxicity in 1
Kosmider et al. (33)	19	Retrospective	Y-90 radioembolization *plus* 5-fluorouracil and leucovorin (*n* = 7) or FOLFOX (*n* = 12)	ORR: 84% (2CR, 14PR)	PFS: 10.4 m; OS: 29.4 m; significantly better survival if no EHD (37.8 vs. 13.4 m)	Febrile neutropenia with concurrent FOLFOX treatment, a perforated duodenal ulcer, and one death from hepatic toxicity

*CR, complete response; PR, partial response; SD, stable disease; PD, progressive disease; PFS, progression free survival; EHD, extra-hepatic disease; ORR, objective response rate; OS, overall survival; CEA, carcinoembryonic antigen; TTP, time to progression; HAC, hepatic arterial chemoinfusion*.

A subsequent phase 2 study by the same group addressed some of the issues related to the first study (31). Patients were randomized to receive systemic chemotherapy with 5-fluorouracil and leucovorin alone or in combination with a single hepatic artery infusion of Y-90 resin microspheres (Table 2). The results supported the fact that the addition of single infusion of Y-90 microspheres resulted in significantly higher objective response rate (ORR: 90.1 vs. 0%; *p* < 0.001), longer TTP (18.6 vs. 3.6 months; *p* < 0.0005), and better median survival (29.4 vs. 12.8 months; *p* = 0.025). This study was limited by the small patient cohort (total of 21 patients only) and use of 5-FU and leucovorin only for chemotherapy.

Sharma et al., in a phase 1 study, assessed the toxicity and maximum tolerated dose of oxaliplatin during the first three cycles of chemotherapy (FOLFOX) when used as a radiation sensitizer for Y-90 therapy that was administered within 3–4 days of first cycle of chemotherapy (Table 2) (32). Patients received standard dose oxaliplatin from the fourth cycle of chemotherapy (FOLFOX). Most of the patients with bilobar disease received sequential lobar infusion of Y-90 microspheres. The maximum tolerated dose was 60 mg/m^2^ of oxaliplatin for the first three cycles, with full dose FOLFOX thereafter. The toxicity was limited to grade 3 abdominal pain in five patients, Grade 3–4 neutropenia in 12 and one episode of Grade 3 hepatotoxicity. They reported a 90% partial response (PR) rate at 12 weeks, median progression free survival (PFS) of 9.3 months and TTP of 12.3 months. In patients with no extra-hepatic disease, the PFS was 14.2 months. This study is significant in that it demonstrated the safety of combining Y-90 therapy with standard first line systemic chemotherapy (FOLFOX). Since it is a phase I study without randomization to a chemotherapy alone arm, it is difficult to draw conclusions on the effectiveness of such combination therapy. The median PFS and overall survival reported by the authors who applied FOLFOX regimen used as first line chemotherapy without Y-90 radioembolization range from 7.6 to 9.0 months and 16.2 to 19.5 months, respectively.

In a retrospective review, Kosmider et al. reported results of combination therapy with Y-90 microspheres and systemic chemotherapy with FOLFOX or 5-FU + leucovorin as first line therapy for hepatic metastases from colon cancer (Table 2) (33). The reported objective response rate was 84% with a median PFS of 10.4 months and median survival of 29.4 months. The median survival was significantly better in patients with no extra-hepatic disease (37.8 vs. 13.4 m). This study is limited by small number (only 19 patients), retrospective nature, and varied chemotherapy regimens used.

In summary, Y-90 therapy in combination with first line chemotherapy is safe and may improve tumor response rates. Although a trend toward improved overall survival is reported, more data is required to establish its role in improving overall survival outcomes. There are a few large ongoing clinical studies (SIRFLOX trial:NCT00724503 and FOXFIRE trial:ISRCTN83867919) that include current standard of care chemotherapy regimens (with biological agents) with Y-90 and may help establish the benefit of Y-90 as part of first line therapy.

### Yttrium-90 radioembolization in combination with second- or third-line chemotherapy

Van Hazel et al. reported results of concomitant therapy of Y-90 radioembolization with systemic irinotecan in patients who failed initial 5-FU chemotherapy (Table 3) (34). Irinotecan was administered to assess maximum tolerated dose. In a group of 25 patients, 11 (48%) patients had PR and 9 (39%) had stable disease (SD). The median overall survival was 12.2 months. Grade 3–4 toxicities occurred in 12 (48%) patients.

**Table 3:** **Yttrium-90 radioembolization in combination with second- or third-line chemotherapy**.

Author	No. of patients	Trial design	Treatment	Response	Survival	Complications
Van Hazel et al. (34)	25	Prospective, dose escalation study	Irinotecan at 50, 75, or 100 mg/m^2^ on days 1 and 8 of a 3-week cycle for the first two cycles, and full irinotecan doses (i.e., 100 mg/m^2^) during cycles 3–9. Radioembolization during the first chemotherapy cycle	PR in 11 (48%) of 23, and SD in 9 (39%)	Median PFS: 6.0 months; Median OS: 12.2 months	Grades 3–4 events in three of six patients at 50 mg/m^2^ (obstructive jaundice, thrombocytopenia, and diarrhea), in five of 13 patients at 75 mg/m^2^ (neutropenia, leukopenia, thrombocytopenia, elevated alkaline phosphatase, abdominal pain, ascites, and fatigue) and in four of six patients at 100 mg/m^2^ (diarrhea, deep vein thrombosis, constipation, and leukopenia)
Lim et al. (35)	30	Prospective; all patients who failed initial 5-FU chemotherapy, 22 failed oxaliplatin or irinotecan also. EHD in 7	Radioembolization; concurrent 5FU in 21	PR in 10 (33%); SD in 8 (27%); no response in patients with poor performance status or with extra hepatic disease	TTP: 5.3 months	Duodenal/gastric ulcer in 13%. One death related to radiation hepatitis

*CR, complete response; PR, partial response; SD, stable disease; PD, progressive disease; PFS, progression free survival; EHD, extra-hepatic disease; ORR, objective response rate; OS, overall survival; CEA, carcinoembryonic antigen; TTP, time to progression; HAC, hepatic arterial chemoinfusion*.

In another study, Lim et al. reported results from a heterogeneous group of patients (*n* = 30) who were treated with 5-FU as first line chemotherapy (Table 3) (35). Eight of these patients failed 5-FU, 14 patients failed 5-FU and subsequent oxaliplatin and irinotecan, and 8 patients failed 5-FU and subsequent oxaliplatin or irinotecan. Thirty-three percent had PR and TTP was 5.3 months. There was 13% incidence of duodenal/gastric ulcers from non-target embolization of Yttrium-90 microspheres.

### Yttrium-90 radioembolization as a salvage treatment for chemotherapy-refractory disease

Multiple retrospective studies reported outcomes when Y-90 was used as salvage therapy in chemorefractory patients (Table 4) (19, 21, 22, 36–40). Bester et al. reported the results of a retrospective study to evaluate the safety and survival of patient with chemotherapy-refractory metastatic CRC to the liver treated with resin microsphere Y-90 (*n* = 224) compared with patients who underwent standard/supportive care (*n* = 51) (Table 4) (39). The median overall survival after radioembolization was 11.9 months compared to 6.3 months for the standard care cohort. The incidence of duodenal/gastric ulceration was 3.2%.

**Table 4:** **Yttrium-90 as salvage therapy for chemorefractory patients**.

Author	No. of patients	Trial design	Treatment	Response	Survival	Complications
Kennedy et al. (19)	506	Retrospective, multi-center; extra-hepatic disease in 35%; 90% received prior chemotherapy and 30% received liver surgery or ablation	Radioembolization with Y-90		Median OS: 10.1 months	Total Grade 1–3 events: 32% gastrointestinal events, 44% fatigue, and 1% liver failure
Kennedy et al. (21)	208	Retrospective, multi-center; 100% failed first line chemotherapy (FOLFOX ± Avastin/Erbitux), 94% failed second line (FOLFIRI ± Avastin/Erbitux), and 87% failed third line chemotherapy (Capecitabine ± Avastin/Erbitux); 46% had liver-directed therapies	Radioembolization with Y-90		Median OS 10.5 months for responders and 4.5 months for non-responders	Computed tomography partial response was 35%; positron emission tomography response of 91%
Cianni et al. (36)	41	Retrospective; all patients progressed on several systemic chemotherapy regimens	Radioembolization with Y-90	CR in 2, PR in 17, SD in 14, and PD in 8 patients	Median OS: 354 days, median PFS: 279 days	One Grade 4 hepatic failure, two Grade 2 gastritis, and one Grade 2 cholecystitis
Hendlisz et al. (37)	46 (44 Analyzed)	Prospective, multi-center, and randomized study	Patients who failed systemic chemotherapy were randomized to receive 5FU (26) or 5FU plus Y-90 (24)		Median TTLP was 2.1 vs. 5.5 m; median TTP was 2.1 vs. 4.5 m; median OS: 7.3. 10 m	No significant toxicities in the group, which received 5FU and Y-90
Cosimelli et al. (38)	50	Multi-center, phase II prospective study; 38 patients had ≥4 lines of chemotherapy	Radioembolization with Y-90	CR in 1, PR in 11, SD in 12, and PD in 22.	Medina OS 12.6 m; improved survival in patients who responded to Y-90 (16 vs. 8 m)	Two deaths in 2 months
Bester et al. (39)	224 vs. 51	Retrospective case control study; Y-90 vs. best supportive care for chemorefractory liver metastases	Radioembolization with Y-90 vs. best supportive care		Median OS 11.6 vs. 6.3 m	11 Cases (3%) of ulceration, 10 cases (2.9%) of radiation-induced liver disease, and 6 complications (1.8%) involving the gallbladder (e.g., cholecystitis)
Evans et al. (40)	140	Retrospective study; 35% extra hepatic disease	Radioembolization with Y-90		Median OS 7.9 months	
Saxena et al. (22)	979	Systematic review of 20 studies; patients afield median 3 regimens of chemotherapy		CR: 0%, PR: 31%; SD: 40.5%	Median TTLP 9 months. Median OS 12 months	Mostly Grade 1 and 2 toxicities

*CR, complete response; PR, partial response; SD, stable disease; PD, progressive disease; PFS, progression free survival; EHD, extra-hepatic disease; ORR, objective response rate; OS, overall survival; CEA, carcinoembryonic antigen; TTP, time to progression; TTLP, time to progression in the liver; HAC, hepatic arterial chemoinfusion*.

In a phase II multi-centered clinical trial for chemotherapy-refractory colorectal liver metastasis, Cosimelli reported an overall response rate of 24% using the RECIST criteria (38). The median time to progression was 3.7 months and the median overall survival was 12.6 months. Apart from two deaths at 40 days (from renal failure) and 60 days (liver failure) after treatment, all of their adverse events were classified within the WHO Grade 1/2 category. Two patients from their cohort were downsized with radioembolization to enable potential curative resection.

A recent systematic review by Saxena et al. (*n* = 979) reported a median time to intrahepatic progression of 9 months and a median overall survival of 12 months, the overall acute toxicities ranged from 11 to 100% (median 41%) with most of the cases being mild – Grade 1/2 (median 39%), which resolved without intervention (22).

Overall, median survival is in the range of 10–12 months in these studies. The response rates are at 31% PR and 41% SD (22). Presence of large volume disease (>25% of liver volume), extra-hepatic disease, poor response to Y-90, and ≥3 lines of prior chemotherapy portend poor prognosis (22). Given that the median survival of patients who failed third line chemotherapy is in the range of 4–6 months, therapy with Y-90 may improve survival outcomes. The utility of concomitant chemotherapy needs to be further explored.

## Recommendations

Yttrium-90 therapy is recommended for chemorefractory patients with liver-only or liver-predominant disease and in patients who do not wish to have systemic chemotherapy. Use of Y-90 therapy in conjunction with standard first line or second line chemotherapy requires more rigorous data and is recommended in a clinical trial setting. The use of Y-90 is not recommended in patients with extensive extra-hepatic disease or extensive bilobar hepatic involvement. Similarly, patients with poor performance status (ECOG PS >2) are not suitable for Y-90 therapy.

## Conclusion

Yttrium-90 microsphere therapy is being increasingly used for treatment of colorectal metastases to the liver. Patients who have failed systemic chemotherapy appear to benefit from this therapy. Concurrent use of Y-90 in first and second line chemotherapy is currently being investigated. Future trials need to focus on identifying specific target populations who may benefit most from this therapy.

## Conflict of Interest Statement

The authors declare that the research was conducted in the absence of any commercial or financial relationships that could be construed as a potential conflict of interest.
